# Precautions against Plague

**Published:** 1899-09-09

**Authors:** 


### PRECAUTIONS AGAINST PLAGUE.

It is very satisfactory to hear of the precautions which are being taken by the Local Government Board with the object of lessening the chance of plague being imported into this country. We are told that the various ports which might become points of entry have been inspected, and that all is well, which is in every way a thing to be thankful for. It may be worth while, however, to recall the fact that the organisation made by the port sanitary authorities, which is what has recently been inspected, is one which has for its object the discovery and isolation of such cases as may arrive on our shores; that its provisions were devised with the special object of dealing with cholera, which is mainly a water-borne disease, and one which tends to die out rapidly wherever the water supply is well protected; and that we have no proof whatever of the efficiency of these arrangements in holding in check such an infection as that of plague; so that, after all, gratifying as it is to learn that the Local Government Board and the port sanitary authorities are on the alert, we see therein no proof of our security. No doubt the arrangements made at our ports were fairly successful in preventing cholera—a disease of short incubation—from obtaining a footing in England. Still, they failed in several instances, and cholera got through the cordon in a sufficient number of cases to show that England's comparative immunity was due to its improved internal sanitary condition, especially in regard to water supply, rather than to the greater vigilance of its port sanitary authorities. But even if our port arrangements had proved completely effectual against cholera, that would mean but little in regard to plague, a disease which certainly has nothing to do with water.

If it is claimed that our sanitary organisation is capable of exercising an effectual control over plague, a disease regarding whose mode of spread we know practically nothing, it is fair to ask what about scarlet fever, concerning which we profess to know a good deal, and diphtheria, about whose incomings and outgoings our bacteriologists seem to know every detail, and against whose attacks we possess an immunising and antitoxic serum of great efficacy. The first of these attacked upwards of 16,000 persons last year in London alone, while, as to the latter, in spite of all our knowledge, our sanitary precautions, and our isolation hospitals, its prevalence in London has more than doubled within the past ten years. In the face of such facts as these it is idle to talk of infection being brought under control, and we have to face the uncomfortable fact that if the conditions which are favourable to an infectious disease exist the malady will take root among us, while if they don't they won't. There is the whole question in a nutshell. The whole question then is, Do the conditions exist which would allow plague to take root were it to be introduced, as it certainly would be were it to prevail just over the water?

So far as the great mass of English people are concerned we have no hesitation in saying that they need not worry about plague. But there are spots where, without doubt, the conditions exist which are known to be those amid which plague spreads when it takes root; and this is as much as can be said on the subject.

When, then, we hear talk of keeping plague at bay by medical inspection and by isolation we must consider how other diseases are kept at bay. The practical suppression of typhus is a triumph of sanitary science. But typhus vanished not because the cases were isolated, but because the conditions in which it could flourish were removed; typhoid has been diminished not by isolation but by improving the water supply, and where the water supply has not been improved there it persists; cholera has failed to take root during recent prevalences, not because the cases were isolated, but because the conditions necessary for its spread had been eliminated. Meanwhile scarlet fever, measles, and whooping-cough persist, presumably because the conditions fitted for their development have not been removed, and diphtheria actually increases in response to changes in modern social conditions, notwithstanding all that has been done to stop it by isolation. These things must not be forgotten. Cholera killed many people, but it did an immense service to England by forcing us to protect our water supplies. Let us hope that plague, even without killing many people, will do us the same service in regard to other sanitary defects, and that its approach will strengthen the hands of those who fight against the dirt, the overcrowding, and the verminous conditions which characterise too many of our slums, conditions which are well known to be those amid which plague flourishes the best.

